# Artificial intelligence model based on CT imaging for predicting infected upper urinary tract calculi

**DOI:** 10.3389/fsurg.2026.1663253

**Published:** 2026-04-15

**Authors:** Shichao Song, Tao Ma, Jiandong Wang, Yonggang Li, Zhu Wang, Wenzeng Yang, Zhenyu Cui

**Affiliations:** 1 Department of Urology, Affiliated Hospital of Hebei University, Baoding, Hebei, China; 2 Department of Urology, The Third Hospital of Bazhou City, Langfang, Hebei, China

**Keywords:** artificial intelligence, deep learning, infectious stones, stone composition, urinary tract calculi

## Abstract

**Objective:**

To construct an artificial intelligence (AI) model based on Computed Tomography (CT) imaging and evaluate its efficacy in preoperatively predicting infected upper urinary tract calculi.

**Methods:**

Clinical data from December 2023 to February 2025 for patients diagnosed with urinary tract calculi at the Affiliated Hospital of Hebei University were collected. Postoperative analysis of stone composition defined stones containing more than 25% struvite and/or carbonate apatite as infectious stones, with the remainder being non-infectious stones. Labelimg software was utilized to annotate the stone locations in CT images by manually outlining the stone contours. Stratified random sampling was performed at the patient level to divide the 465 enrolled patients into training, validation, and test sets at a 7:1:2 ratio (326, 47 and 92 patients, respectively), with all CT images of each patient assigned to the corresponding dataset to avoid data overlap. We documented the model's Average Precision (AP), Mean Average Precision (mAP), and Mean Recall (mR). Additionally, CT images from patients diagnosed with urinary tract calculi from December 2021 to February 2023 at our hospital were randomly selected to evaluate the model's clinical efficacy.

**Results:**

Of the 465 patients enrolled, 134 were classified in the infectious stone group and 331 in the non-infectious stone group. The model's mAP for infectious stones in the training and validation sets was 95.3% and 95.0%, respectively. The mAP was lower at 62.4% for stones smaller than 32 × 32 pixels, and 81.3% for stones larger than this size. Of the 935 CT images analyzed from December 2021 to February 2023, the RetinaNet model achieved an accuracy of 85.17%, sensitivity of 72.78%, specificity of 93.09%, and positive and negative predictive values of 87.04% and 84.27%, respectively for predicting infectious stones. The kappa test demonstrated significant consistency between the model and infrared spectroscopy analysis (kappa value of 0.679).

**Conclusion:**

The RetinaNet model based on CT imaging shows high specificity for predicting infectious upper urinary tract calculi, supporting its clinical value in identifying suspected cases preoperatively. However, its moderate sensitivity precludes reliable standalone ruling-out of infectious stones. When combined with routine laboratory tests (e.g., urine routine and culture), this AI model acts as a valuable complementary preoperative tool, providing auxiliary guidance for treatment strategy formulation and surgical decision-making in patients with urinary tract calculi.

## Introduction

Urinary tract calculi represent a pervasive global health challenge, with a worldwide prevalence ranging from 5% to 10% (1, 2). These calculi are categorized into infectious and non-infectious types, wherein infectious stones constitute approximately 15% of cases. Predominantly composed of magnesium ammonium phosphate hexahydrate, these stones harbor a significant population of urease-producing bacteria. These stones grow rapidly, can cause severe renal impairment, and are more likely to induce severe complications (e.g., pyelonephritis) with a higher recurrence rate. If left untreated, they are associated with a high mortality rate (3). Consequently, aggressive surgical intervention is imperative upon diagnosis.

The formation mechanisms of urinary tract calculi are multifaceted, involving factors such as supersaturation-induced crystallization and crystal-mediated renal injury (4, 5). The genesis of infectious calculi is intimately associated with urease-producing bacteria, wherein the pH alterations stemming from urea decomposition play a pivotal role. Presently, the diagnostic approach for urinary tract calculi relies on imaging examinations such as ultrasound, Kidney, Ureter, Bladder (KUB), Intravenous Urography (IVU), and CT, alongside laboratory investigations including blood, urine, and stone composition analysis. Among these, CT emerges as the preferred diagnostic modality, owing to its superior sensitivity and accuracy. Conventional CT attenuation value analysis is simple but limited by density overlap and measurement errors, only enabling rough prediction of stone composition (6). Although dual-energy CT has greatly improved the specificity of uric acid stone detection, it still faces technical bottlenecks in subclassifying non-uric acid stones, diagnosing tiny stones, and quantifying complex mixed stones (7). Notably, stone composition analysis is usually performed postoperatively, leading to diagnostic delay. During surgical intervention for infectious stones, there is a risk of bacteremia, potentially leading to severe infections. Consequently, the selection of an appropriate surgical technique is of paramount importance. For large stones, staghorn calculi, and those that cannot be managed via a retrograde approach, percutaneous nephrolithotomy (PCNL) is the preferred surgical modality. However, the risk of postoperative complications cannot be overlooked (8). Accurate preoperative diagnosis of infectious stones is essential for personalized patient management, enabling the selection of an appropriate surgical approach and the mitigation of complications.

AI, by mimicking human learning processes, possesses the potential to exceed human intellectual capabilities (9). As a core component of AI, machine learning (ML) excels in handling extensive clinical datasets, thereby accumulating experience and refining diagnostic accuracy. Deep learning (DL), a subset of ML, autonomously extracts pixel-level features from images, minimizing manual feature selection and demonstrating significant advantages in medical imaging and pathology analyses (10).

The integration of AI with the medical domain is propelling advancements in medical research and clinical practice. Leveraging extensive clinical datasets, AI empowers physicians to predict diseases more accurately, thereby enhancing operational efficiency, refining treatment strategies, and curbing unnecessary expenditures. AI has achieved progress in diagnosing and treating breast cancer, dermatological conditions, neuro-oncology, and cardiovascular disorders. In the field of urology, AI is increasingly showcasing its potential. For instance, Kazemi et al. (11) employed ensemble learning models to predict stone characteristics, Kriegshauser et al. (12, 13) analyzed the relationship between CT images and stone composition using AI, while Parakh et al. (14) developed a highly accurate automated diagnostic model. Presently, AI research in the context of urinary stones mainly centers on the prediction of specific compositions. This study, however, aims to pioneer a model dedicated to the prediction of infectious stones. This study aims to develop a predictive model for infectious stones using AI technology to analyze preoperative CT images and stone composition reports, thereby constructing a predictive model for infectious stones and evaluating its clinical efficacy. AI-based predictions hold the potential to control stone recurrence, optimize surgical strategies, and mitigate infection risks and recurrence rates.

## Materials and methods

### Study population

This retrospective study analyzed the clinical data of 465 patients diagnosed and treated for urinary tract calculi at the Affiliated Hospital of Hebei University between December 2023 to February 2025. Stratified random sampling was performed at the patient level to divide the 465 enrolled patients into training, validation, and test sets at a 7:1:2 ratio (326, 47 and 92 patients, respectively), with all CT images of each patient assigned to the corresponding dataset to avoid data overlap. These datasets were utilized for the training of the RetinaNet model and the comprehensive evaluation of its performance. Furthermore, we selected urinary tract CT scans from patients treated at the Affiliated Hospital of Hebei University between December 2021 and February 2023 to evaluate the model's clinical efficacy. The stone size for all cases was 14.8 ± 5.7 mm, with a minimum stone diameter of 4.2 mm, including 21 cases of ureteral stones that were passed spontaneously. The study was approved by the Institutional Ethics Committee of Affiliated Hospital of Hebei University (No. HDFYLL-KY-2024-087; date:March 27th, 2024).

Inclusion criteria: (1) Patients who underwent pre-treatment non-contrast CT scans of the urinary tract at our hospital, yielding stone-related CT images; (2) Patients who underwent post-treatment infrared spectroscopic analysis of stones at our hospital, obtaining stone composition results; (3) Patients with clear CT images, devoid of motion artifacts or other interferences, and with unobstructed visualization of the stone locations.

Exclusion criteria: (1) Patients who did not undergo pre-treatment non-contrast urinary tract CT scans or only underwent contrast-enhanced CT scans, resulting in unavailable non-contrast images; (2) Patients who did not undergo post-treatment infrared spectroscopic analysis of stones, resulting in unavailable compositional results; (3) Patients with CT images that were either unclear or exhibited motion artifacts or other interferences; (4) Patients whose CT images revealed the presence of indistinguishable foreign objects, such as DJ stents or catheters, at the stone locations; （5）Pregnant women with urinary tract calculi; (6) Patients with incomplete or missing clinical data.

### Image filtering, annotation, and segmentation

**Figure 1:**
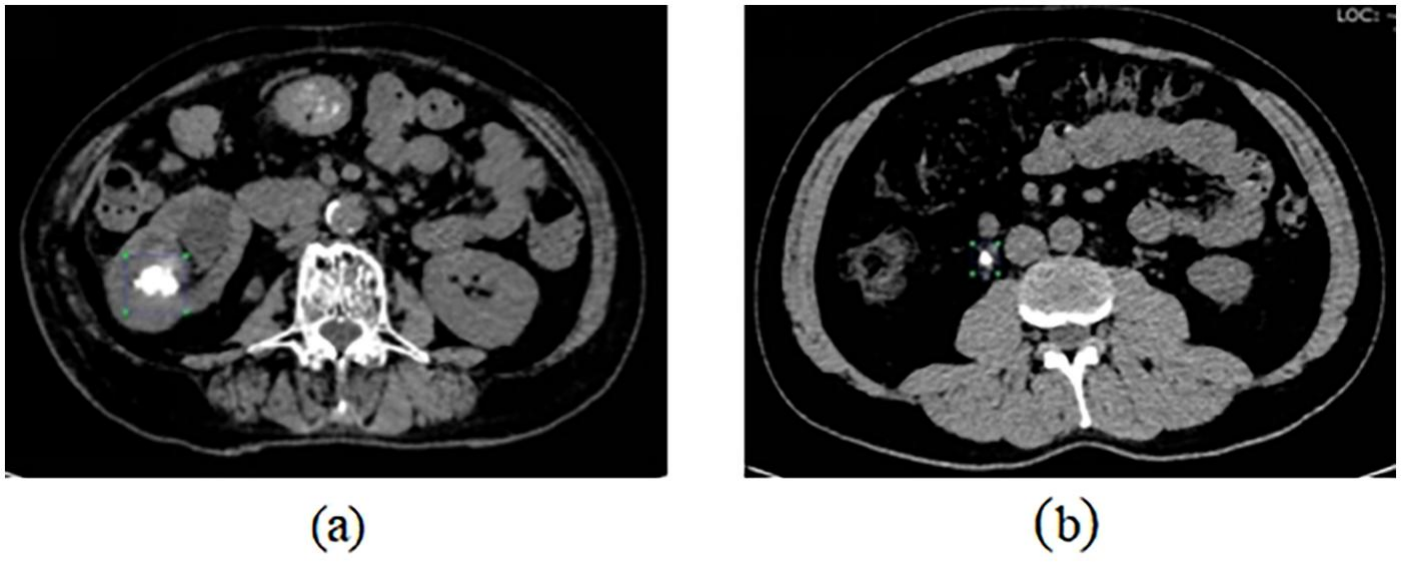
Annotation of stone regions in CT images using labelimg. **(a)** indicates kidney stone annotation, **(b)** indicates ureteral stone annotation.

Images with prominent stone regions from non-contrast urinary tract CT scans were selected and annotated. The labelimg tool (V1.8.1) was employed to delineate the stone regions in each CT image, designating them as regions of interest (ROIs), as illustrated in Figure 1. Before formal annotation, all annotators received unified training and calibration by senior urologists and radiologists. A standardized annotation protocol was formulated and strictly implemented. Two independent senior investigators rechecked a random subset of annotated images to assess consistency; inconsistent annotations were collectively discussed and revised to ensure accurate and uniform image labeling.

Infrared spectroscopic analysis facilitates the quantitative detection of stone compositions within specimens, including carbapatite, magnesium ammonium phosphate hexahydrate, calcium oxalate monohydrate, and anhydrous uric acid. Based on the quantitative analysis report, stones with a total composition exceeding 25% of magnesium ammonium phosphate hexahydrate and/or carbapatite are classified as infectious stones, whereas the remainder are designated as non-infectious stones (15).

### Model construction

A RetinaNet model was constructed to investigate predictions of stone infectiousness based on CT imaging. Performance metrics of RetinaNet include AP, mAP, and mR, with values approaching 100% signifying superior model performance. The dataset comprised a total of 13,275 CT images from 465 patients, including 2,782 images from 134 patients with infectious stones and 10,493 images from 331 patients with non-infectious stones.

### Network model selection and training

CT data from each phase of the training group were individually input into the Mobilenetv3-large network for training purposes, constructing non-contrast, arterial, venous, excretory, and all-phase combined models separately. The training process for all networks employed the Adam optimizer coupled with a cross-entropy loss function. The finalized hyperparameters for model training were set as follows: learning rate of 0.0001, batch size of 16, and a total of 100 optimization iterations.

The models were created using the Python 3.7 programming language and were compiled and trained using PyTorch 1.10 and CUDA 11.2 on a desktop workstation equipped with an RTX 3060 GPU. The Jupyter software served as the integrated development environment for the modeling process.

### Network model testing and optimal model screening

The experimental environment was established on an Ubuntu 20.04 system, wherein the experiments were carried out using Python 3.8, PyTorch 1.10.0, and CUDA 11.3. The NVIDIA GeForce 2080Ti GPU served as the IDE tool. The CT images of stones were acquired in BMP format, each with a resolution of 512 × 512 pixels. For model parameter optimization, gradient descent was employed, with a batch-size of 4, an initial learning rate during training of 0.005, a weight decay coefficient of 0.0005, and a momentum of 0.9. The training process was iterated over 50 epochs.

### Clinical validation of the optimal model

A random subset of CT images from 87 clinical patients with urinary tract calculi was selected, comprising 49 patients with infectious stones and 38 patients with non-infectious stones, yielding 365 and 570 images for each group, respectively. These images were input into the RetinaNet network model, and the model's predictions were compared against the results from a stone analyzer. The clinical performance of the model was assessed using metrics including accuracy, sensitivity, specificity, positive predictive value, negative predictive value, and kappa coefficient.

### Statistical analysis

Utilizing Python 3.8, we computed the AP, mAP, and mR metrics for the RetinaNet network model across the training, validation, and testing datasets. In the clinical diagnostic assessment of infectious stones, IBM SPSS Statistics 26 was employed to analyze and determine the model's accuracy, sensitivity, specificity, positive predictive value, and negative predictive value. Additionally, the kappa coefficient was calculated using IBM SPSS Statistics 26 to evaluate the consistency between the RetinaNet network model and infrared spectroscopy analysis.

## Results

**Figure 2:**
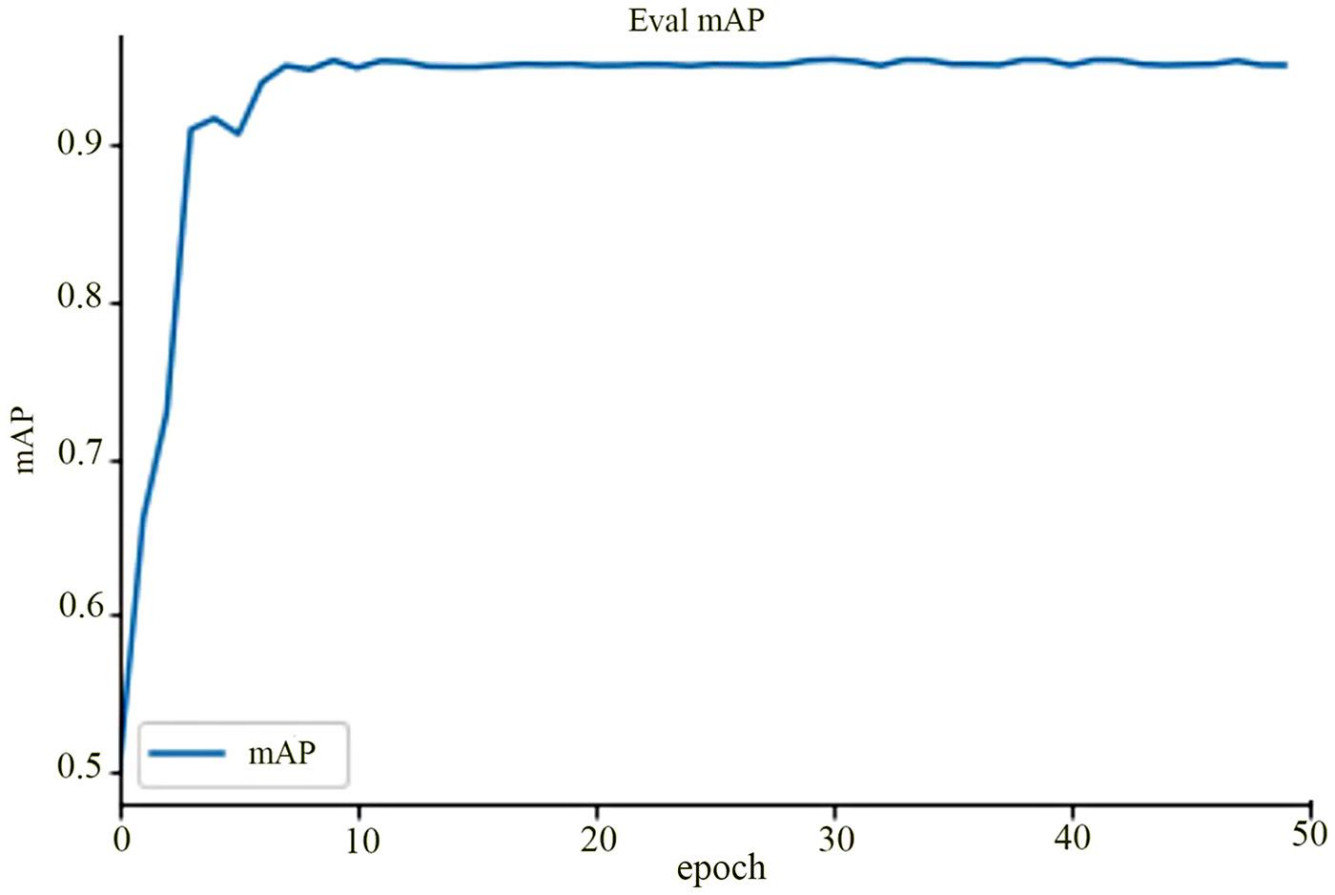
mAP curve for the training set (shows a gradual increase in mAP as training epochs progress, stabilizing at 95.3 after 50 epochs).

**Figure 3:**
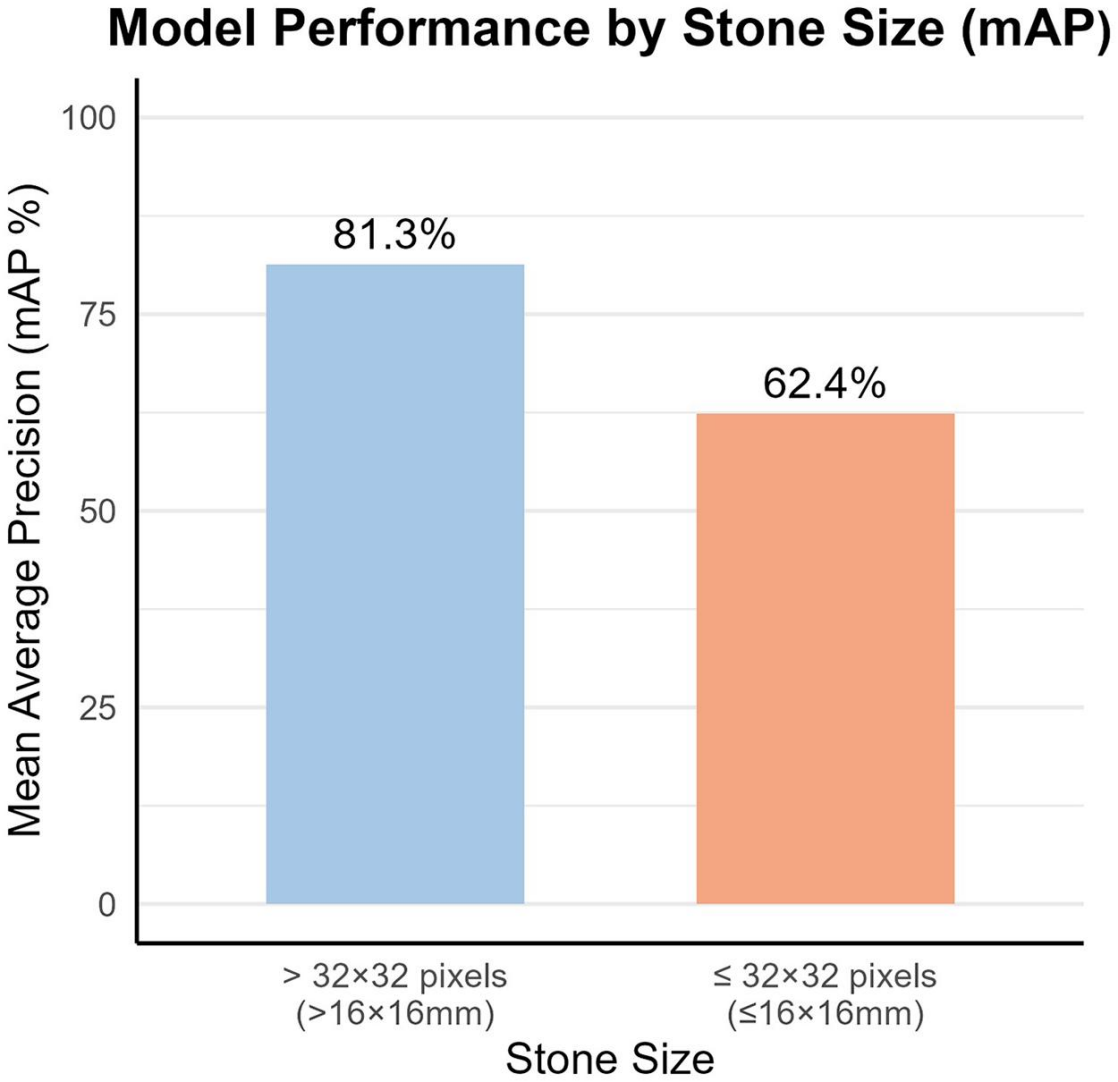
Bar chart showing the performance of the retinaNet model in predicting infectious stones stratified by stone size. The mean average precision (mAP) for stones larger than 32 × 32 pixels (>16 × 16 mm) was 81.3%, compared to 62.4% for stones ≤32 × 32 pixels (≤16 × 16 mm), indicating superior model performance for larger stones.

A total of 13,275 CT images of patients with urinary calculi were collected for constructing the RetinaNet model. The performance parameters of the model across the training, validation, and test sets are outlined in Table 1, while the mAP curve graph for the training set is shown in Figures 2, 3.

**Table 1:** Performance parameters of the retinaNet model.

Dataset	Image count	Stone composition	Stone size (mm)	Image count	AP (%)	mAP (%)	mR (%)
Training Set	9,536	Infectious Stone	15.2 ± 5.9	1,968	98.4	95.3	78.5
		Non-Infectious Stone	14.5 ± 6.1	7,568	98.6		
Validation Set	1,327	Infectious Stone	13.9 ± 5.4	297	93.2	95.0	70.9
		Non-Infectious Stone	16.1 ± 5.3	1,030	96.7		
Test Set	2,412	Infectious Stone	14.6 ± 6.3	517	94.3	94.3	71.4
		Non-Infectious Stone	15.5 ± 5.8	1,895	97.7		

The model's detection performance exhibited variability depending on the stone size. The mAP values, delineated by a size threshold of 32 × 32 pixels, are presented in Table 2.

**Table 2:** Detection performance parameters the model based on stone size.

Stone Size^a^	mAP (%)
≤ 32 × 32 pixels	62.4
> 32 × 32 pixels	81.3

a 32 × 32 pixels (corresponding to ≤16 × 16 mm, based on the CT scan parameter of 0.5 mm/pixel in our study).

**Figure 4:**
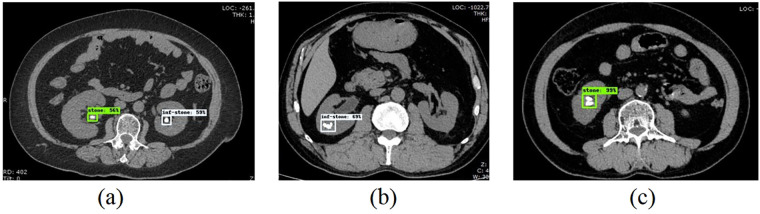
Example prediction images (inf-stone denotes infectious stones, stone denotes non-infectious stones, subsequent figures indicate probability: **(a)** includes both infectious and non-infectious stones predictions; **(b)** indicates infectious stone prediction; **(c)** indicates non-infectious stone prediction).

A cohort of 935 CT images from patients with urinary calculi was selected, comprising 365 images from patients with infectious stones and 570 from those with non-infectious stones. Predicted results obtained from the RetinaNet model processed images reveal that 11 out of the 935 images demonstrated predictions containing both infectious and non-infectious stones (Figure 4). Potential explanations for this phenomenon are as follows: (1) Coexistence of infectious and non-infectious stones in the same patient; (2) Stone analysis specimens may only partially represent all stones in the patient; (3) The model's predictive capability needs further optimization. These 11 cases with ambiguous mixed predictions were first classified as predictive errors (consistent with real-world clinical practice, where ambiguous diagnostic results are deemed non-diagnostic or erroneous) and included in the full analysis for transparency. A comparative analysis was then performed for both the full dataset (935 images, including the 11 mixed-prediction cases) and the filtered dataset (924 images, excluding these cases). For the full dataset (365 infectious, 570 non-infectious images), the model predicted 262 true positives, 103 false negatives, 44 false positives, and 526 true negatives. For the filtered dataset (360 infectious, 564 non-infectious images), the predictions categorize 262 images as infectious stones and 98 as non-infectious stones within the infectious stone group; 39 images are labeled as infectious stones and 525 as non-infectious stones within the non-infectious stone group, as detailed in Table 3.

**Table 3:** Stone prediction results using the model in clinical settings.

		Infrared spectroscopy		Total
Model	Group	Infectious	Non-infectious	
RetinaNet Model Prediction	Infectious	262	39	301
	Non-Infectious	98	525	623
Total		360	564	924

In accordance with the model's predictions derived from the CT images, statistical analyses yielded metrics including sensitivity, specificity, positive predictive value, and negative predictive value, which are presented in Table 4.

**Table 4:** Statistical analysis of prediction results in clinical settings.

Group	Images (*N*)	Sensitivity (%)	Specificity (%)	Positive predictive value (%)	Negative predictive value (%)	Accuracy (%)
Infectious	360	72.78	93.09	87.04	84.27	85.17%
Non-Infectious	564	93.09	72.78	84.27	87.04	

We employed the kappa test to assess the consistency between the RetinaNet network model and infrared spectroscopy analysis, resulting in a kappa coefficient of 0.679, with a 95% confidence interval of 0.630–0.728. Kappa values are classified as indicating slight agreement (0.01–0.20), fair agreement (0.21–0.40), moderate agreement (0.41–0.60), substantial agreement (0.61–0.80), and near-perfect agreement (0.81–0.99). Consequently, a substantial agreement between the RetinaNet network model and infrared spectroscopy analysis is established.

## Discussion

The personalized management of urinary calculi is significantly enhanced by the analysis of stone composition, which is broadly categorized into chemical and physical methods (16). Chemical analysis was historically used but limited by complex procedures and large sample requirements. In contrast, physical methods—especially infrared spectroscopy—have become the clinical gold standard due to high sensitivity, precision, and minimal sample requirements. Studies highlight females and elevated urinary pH as predisposing factors for infectious stones. Although efforts have been made to predict stone composition using CT values, the accuracy of such predictions remains limited. A study by Ye et al. (17) involving nearly 50,000 urinary calculi patients demonstrated a higher prevalence of calcium oxalate and uric acid stones in males, whereas carbonate apatite and struvite stones were more commonly observed in females. Additionally, Yongzhi et al. (18) identified a higher incidence of urinary tract infections in female patients with urinary calculi compared to males. The shorter and straighter female urethra predisposes them to persistent or recurrent UTIs, leading to a higher likelihood of infectious stone formation than in males. Studies have shown that patients with infectious stones have significantly higher rates of postoperative fever and residual stones. Preoperative prediction helps in selecting a more thorough stone clearance technique (such as PCNL) to remove the infection source and in adopting early, longer-course antibiotic strategies during the perioperative period, thereby effectively reducing the risk of infectious complications (19). The challenge of preoperatively predicting stone composition to effectively slow stone progression has garnered extensive research attention. This study leverages AI and the RetinaNet model to predict stone infectivity through CT imaging, presenting an innovative approach for preoperative management.

A multitude of researchers are dedicated to employing CT imaging for the preoperative prediction of stone composition. Some have attempted to correlate stone analysis with urinary CT, comparing CT values of various stone compositions but found the differences inadequate for reliable predictions (20, 21). In contrast, Li et al. (22) demonstrated that average CT values hold promise in predicting calcium oxalate and uric acid stones. Shunhavanich et al. (7) further refined prediction accuracy by incorporating both the mean and standard deviation of CT values. Dual-energy CT has exhibited superior performance in stone composition prediction, a finding substantiated by Jung et al. (23) in their efficacy studies on uric acid stone detection. Nonetheless, current research primarily concentrates on calcium oxalate and uric acid stones, offering limited clinical guidance.

Object detection, a pivotal domain in computer vision, is dedicated to the localization and classification of objects within images. Deep learning (DL)-based object detection algorithms are broadly classified into one-stage and two-stage types; the former offers faster speed with relatively lower accuracy, whereas the latter provides the opposite. RetinaNet, a one-stage network, enhances accuracy through the focal loss function, achieving fast and precise detection. This study employs the RetinaNet model to predict the infectivity of stones using urinary CT images. Infectious stones generally contain struvite and carbonate apatite, but minor amounts of these components do not significantly impact patients clinically. Drawing upon the research of Nevo et al. (15), we defined stones with over 25% of these compositions as infectious. The model's training, validation, and test sets were split in a 7:1:2 ratio, with the evaluation metrics demonstrating RetinaNet's effectiveness in distinguishing infectious stones.

In testing with 935 CT images, the RetinaNet model identified 11 images containing both infectious and non-infectious stones. This phenomenon may be attributed to the simultaneous presence of two stone types in a single patient, incomplete representation of all stones in intraoperative specimen analysis, or insufficient optimization of the model's predictive capability for complex mixed stones. For the original filtered analysis, these 11 cases were excluded to reflect the clinical actionable performance of the model (i.e., only definitive predictions for clinical decision-making), while the full analysis including these cases is now supplemented to ensure research transparency and address potential bias concerns. Notably, the minimal difference in performance metrics between the two datasets confirms that the exclusion of these cases did not artificially inflate the model's efficacy, and the classification of these ambiguous cases as predictive errors aligns with real-world clinical practice where non-definitive AI predictions require further clinical evaluation (e.g., combined with laboratory tests). Statistical analysis of the remaining 924 images revealed a sensitivity of 72.78% and a specificity of 93.09% in predicting infectious stones, alongside positive and negative predictive values of 87.04% and 84.27%, respectively, and an accuracy of 85.17%. The kappa analysis indicated a substantial agreement between the RetinaNet model and infrared spectroscopy, as evidenced by a kappa coefficient of 0.679. These findings indicate the robust performance of RetinaNet in the clinical prediction of infectious stones, thereby providing an invaluable reference for preoperative management and surgical decision-making.

Notably, the RetinaNet model exhibited a marked decline in predictive performance for small stones (≤32 × 32 pixels, 16 × 16 mm; mAP=62.4%), in contrast to the superior performance for larger stones (>32 × 32 pixels, 16 × 16 mm; mAP = 81.3%). This performance discrepancy for small stones is attributable to multiple interrelated factors inherent to CT imaging characteristics and deep learning model design (22). First, small urinary tract stones present with faint pixel-level features on non-contrast CT images, with their attenuation values often overlapping with surrounding renal parenchyma, ureteral wall, or urinary sediment, making it difficult for the model to extract distinct and discriminative features for infectious stone identification. Second, the relatively small sample volume of small infectious stones in the training dataset may lead to insufficient feature learning of the model for this subgroup, resulting in underfitting and reduced predictive accuracy. Third, the default receptive field of the RetinaNet model based on the Mobilenetv3-large backbone is more adapted to the feature extraction of medium and large targets, and it is difficult to capture the fine spatial features of small stones, which further exacerbates the prediction bias.

To address the aforementioned limitations and improve the model's predictive performance for small infectious stones, several targeted technical optimization strategies can be implemented in subsequent research (24–27). First, multi-scale feature extraction modules [e.g., feature pyramid network (FPN) with enhanced top-down fusion, or path aggregation network (PAN)] can be integrated into the RetinaNet backbone. These modules can effectively fuse shallow high-resolution features (rich in small target spatial details) and deep semantic features (rich in stone composition-related feature information), enabling the model to better identify and characterize small stones. Second, small stone-oriented image augmentation strategies should be applied during model training, including random scaling, translation, rotation, and mosaic augmentation of small stone CT images, which can expand the diversity of small stone samples and enhance the model's generalization ability for small target features (28). Third, focal loss function optimization with adjusted hard sample mining weights can be adopted, increasing the loss contribution of small infectious stone samples in the training process to guide the model to focus on learning the feature representation of this easily misclassified subgroup. Fourth, the training dataset can be expanded by incorporating more multi-center small infectious stone CT images, ensuring sufficient sample size for the model to learn the unique features of small stones with different CT attenuation values and morphological characteristics. Additionally, post-processing algorithms [e.g., non-maximum suppression (NMS) with adaptive IoU thresholds for small targets] can be added to the model prediction stage to reduce the false negative rate of small stone detection and improve the overall predictive accuracy for small infectious stones.

The clinical implications of the model's false negatives and false positives are clinically noteworthy. False negatives eliminate perioperative antibiotic prophylaxis, elevating risks of bacteremia, urosepsis and stone recurrence due to unaddressed urease-producing bacteria. False positives may cause unnecessary preoperative antibiotics (fostering resistance) and more invasive surgery, increasing healthcare burden and patient trauma. The model's high specificity (93.09%) minimizes overtreatment, aligning with antimicrobial stewardship, while the lower sensitivity (72.78%) highlights the urgency to reduce false negatives, as their life-threatening risks far outweigh false positive consequences in clinical practice.

This study is not without its limitations. Primarily, its single-center design, with all samples originating from the Affiliated Hospital of Hebei University and constrained by specific CT equipment, may limit the model's generalizability to other medical settings. Future multi-center studies could enhance the model's generalizability. Additionally, as a retrospective study, the findings' conclusiveness may be inferior to that of prospective studies. Furthermore, stone composition analysis relies on infrared spectroscopy, with samples primarily obtained through surgeries, which may introduce sampling errors due to inadequate representation. Finally, the model's performance in detecting smaller stones, such as those in the ureter, remains suboptimal, underscoring the necessity for further refinement. But the limitations and their potential influence on the results would be desirable.

## Conclusion

(1) The RetinaNet CT-based model shows high specificity (93.09%) but moderate sensitivity (72.78%) for predicting infectious calculi: high specificity effectively identifies suspected cases, while moderate sensitivity means it cannot independently rule out infectious stones preoperatively; (2) This AI model is a valuable complementary preoperative tool for urinary tract calculi and should be used with routine laboratory tests (e.g., urine routine, urine culture) to reduce missed diagnoses. Integrated into comprehensive clinical assessment, it provides meaningful auxiliary guidance for individualized treatment strategies and surgical decision-making in such patients.

## Data Availability

The raw data supporting the conclusions of this article will be made available by the authors, without undue reservation.
